# COVID‐19: The heart of the issue

**DOI:** 10.1111/jocs.14684

**Published:** 2020-06-16

**Authors:** Beth Woodward, Muhammed Kermali

**Affiliations:** ^1^ Institute of Cancer and Genomic Sciences College of Medical and Dental Sciences, University of Birmingham Birmingham UK; ^2^ Faculty of Medicine, St. George's, University of London London UK

To the Editor,

We read with great interest the review by Khan et al^1^ who discussed the emerging role of cardiovascular disease and the progression of the novel coronavirus disease 19 (COVID‐19). As part of their review, the authors discussed a broad range of cardiovascular effects being observed during and after infection in this disease. We commend their conclusions that appropriate triage and risk stratification is essential in patients with cardiovascular disease (CVD) and COVID‐19. We encourage the need for further evidence regarding the mechanisms of CVD and particularly the use of therapeutics. This is of particular importance given the global concern about the use of the renin‐angiotensin‐aldosterone system (RAAS) inhibitors in the mechanisms of severe acute respiratory distress syndrome‐coronavirus‐2 (SARS‐CoV‐2).

SARS‐CoV‐2 utilizes the angiotensin‐converting enzyme 2 (ACE2) cell membrane protein on lung alveolar epithelial cells to enter host cells.^1^ This ACE2 enzyme is key in the RAAS cascade so the authors highlighting the importance of understanding this further was justified. RAAS inhibitors contributing to increased severity of COVID‐19, most commonly in hypertension, is a logical hypothesis due to their mode of action. However, it is currently unclear whether this virus's interaction with ACE2 causes dysregulation or interference of downstream effectors. Early observational studies concluded that the use of RAAS inhibitors may not be associated with SARS‐CoV‐2 infection or more severe COVID‐19 progression. One retrospective multicentre study with 1128 participants (188 on a RAAS inhibitor) suggested that in hospitalized COVID‐19 patients with pre‐existing hypertension, RAAS inhibitors had better outcomes compared to non‐RAAS inhibitor use.^2^ This supports continuing their use if already prescribed, but recognizes the significant risk for confounding variables in their methodology.^2^

Discontinuing these medications may potentially put patients at greater risk of complications, directly due to SARS‐CoV‐2 infection or indirectly from a period of blood pressure instability and attenuating their known cardio‐ and renal‐protective effects.^3^ Furthermore, the authors highlighted that cardiovascular complications and myocardial injury have also been observed peri‐ and postinfection with SARS‐CoV‐2, even in patients without pre‐existing CVD.^1^ Given these mechanisms are yet to be elucidated and likely to be multifactorial, this further supports this notion.

We support the conclusion made that there is currently limited evidence to recommend their use should be discontinued prophylactically and in confirmed cases.^3^, ^4^ It is pivotal further preclinical studies are carried out to understand the cardiovascular mechanisms in COVID‐19. We emphasize the need to elucidate mechanisms of RAAS inhibitors in SARS‐CoV‐2 infection. Robust and collaborative human studies are needed to apply this understanding in a meaningful way, where major confounding variables, such as advancing age, can be accounted for.

## CONFLICT OF INTERESTS

The authors declare that there are no conflict of interests.
